# Digital economy and high-quality development of the healthcare industry

**DOI:** 10.3389/fpubh.2024.1331565

**Published:** 2024-01-12

**Authors:** Zijing Ding, Xinyue Qu, Chen Li

**Affiliations:** ^1^School of Economic and Management, Wuhan University, Wuhan, China; ^2^School of Management, Shanghai University of Engineering Science, Shanghai, China

**Keywords:** digital economy, high-quality development, healthcare industry, provinces, China

## Abstract

The high-quality development of the healthcare industry is of great significance for improving people’s health and promoting the construction of a harmonious society. This paper focuses on the relationship between the development of China’s digital economy and the high-quality development of the healthcare industry. Based on the panel data of 30 provinces in China from 2011 to 2020, this paper empirically studies whether the development of the digital economy promotes the high-quality development of the healthcare industry. This study finds that the development of digital economy has significantly promoted the high-quality development of the medical and health industry. The results of this study are still valid after a series of robustness tests including variable substitution, sample adjustment, and endogenous problem mitigation. Heterogeneity analysis shows that the effect of this policy is more significant in the eastern region and southern areas. The results of spatial econometric analysis show that the development of digital economy has obvious spatial spillover effect. The research in this paper can provide reference for developing countries to enhance the development level of digital health industry and improve people’s lives.

## 1 Introduction

The outbreak of COVID-19 in 2020 has greatly affected the safety of people’s lives and property around the world (1–3). In the face of numerous public health emergencies and global population aging trend, the establishment and improvement of medical care system is of great significance and key role for economic growth. On the other hand, the digital wave is sweeping the world (4, 5). With information technology as the core and mobile payment, 5G, cloud computing, and other representatives of the new generation of digital economy (DE), it is reshaping the industrial structure and production mode (6–8), profoundly changing the comparative advantage between countries, and then affecting the global economic and geographical pattern (9).

China is the most populous country in the world, and the aging of the population is obvious. According to the seventh census released by the government of China, the older adult population in China accounts for 13.5 percent of the total, and the older adult dependency ratio is close to 20 percent (10). In the context of high demand for healthcare, the rapidly expanding market demand for healthcare calls for the development of a higher level of healthcare industry, and new opportunities have been ushered in for the development of healthcare industry (11–14). Especially at the dawn of the DE, how the healthcare industry can better apply information technology to the industrial growth, and then promote the high-quality development of the healthcare industry (HDHI). How the development of the DE promotes the transformation and upgrading of the healthcare industry and summarizes the characteristic facts and general laws are the important premise and basis for achieving HDHI.

Previous studies have posited that the integration of technology and healthcare has the potential to enhance the healthcare sector and offer improved and superior services (15). Digital technology has the potential to enhance customer service by leveraging extensive data to better comprehend individual needs and provide tailored experiences (16). Additionally, digital technology’s capacity to disseminate information across temporal and spatial boundaries can serve as a means to reduce social isolation among service recipients, thereby enhancing social integration for marginalized populations (17). The progress in information technology has significant prospects for the healthcare sector, although it also entails certain possible challenges that warrant careful consideration. The field of information technology exhibits a significant level of novelty, necessitating a particular level of group learning ability in order to efficiently acquire mastery. Nevertheless, it is important to acknowledge that the implementation of such measures may result in a “digital divide” phenomenon, particularly impacting certain demographics such as the older adult (18). The progress of the digital health sector is contingent upon the utilization of extensive data for informed decision-making. However, this reliance on data presents a significant obstacle to safeguarding personal privacy and ensuring the security of data assets, particularly within the healthcare domain (19, 20).

Compared to the high quality development-related research that has been published (21–23), this paper argues that high-quality development of healthcare industry (HDHI) has multi-dimensional characteristics. The HDHI is driven by the government and the market, taking innovation, coordination, green, open, and share as the development goals, and aiming at the resources allocation of the healthcare industry. Different from previous studies, this study mainly focuses on the relationship between DE and the HDHI, that is, whether the DE promotes or inhibits HDHI. We further explored the heterogeneous effects that different levels of DE development and geographical locations may have on the HDHI. Due to the possible spatial spillover effect of the development of DE, we further explore this spatial linkage relationship from the perspective of spatial econometrics.

The present study primarily investigates the following three facets. First, the HDHI in China was thoroughly assessed, and the index system was built using four dimensions: medical service, drug service, technical service, and upgrade service. Subsequently, an econometric model is built to determine the correlation between the DE and HDHI. Thirdly, the spatial econometric model is utilized to discover and validate the spatial spillover effect of the development of DE on the HDHI.

## 2 Methods

### 2.1 Benchmark regression model

On the basis of previous research (9), we construct the following econometric model (1) to identify the relationship between DE and HDHI:


(1) $$HDHI_{i,t}=\alpha_{1}DE_{i,t}+\overset{6}{\underset{k=2}{\sum }}\alpha_{k}X_{i,t}+province_{i}+year_{t}+\varepsilon_{i,t}$$

HDHI represents high-quality development of the healthcare industry. DE represents the level of DE development. X is a set of control variables. α is the estimated coefficient, and the coefficient we are most concerned about in this paper is α_1_. province_i_ represents the fixed effect of the province, year_t_ represents the fixed effect of the year, and ε is the random disturbance term.

### 2.2 Quantile regression model

In order to explore the possible differential impact of different levels of DE development on HDHI, we built a quantile regression model in the following form:


(2) $$Q_{\tau }\left(HDHI_{i,t}\right)=\zeta_{1\tau }+\zeta_{2\tau }DE_{i,t}+\overset{7}{\underset{k=3}{\sum }}\zeta_{k\tau }X_{i,t}+\varepsilon_{i,t}$$

In model (2), $Q_{\tau }(HDHI_{i,t})$ is the dependent variable, representing the HDHI at different quantile levels. $\zeta_{2\tau }\cdots \zeta_{7\tau }$ represent the explanatory variables at different quantile levels. Where, $\zeta_{2}$ is the quantile regression coefficient which we are concerned about.

### 2.3 Spatial Durbin model


(3) $$Moran^{'}s\;\;I=\frac{\overset{n}{\underset{i=1}{\sum }}\overset{n}{\underset{j=1}{\sum }}\omega_{ij}\left(x_{i}-\bar{x}\right)\left(x_{j}-\bar{x}\right)}{S^{2}\overset{n}{\underset{i=1}{\sum }}\overset{n}{\underset{j=1}{\sum }}\omega_{ij}}$$

There is a spatial correlation and clustering relationship between the DE and the HDHI. According to existing research (24–27), this paper uses the global Moran index to measure this degree of focus and correlation, so as to reflect its overall characteristics.

In formula (3), $S^{2}$ is the variance of the sample, which is used to represent the degree of dispersion of the sample. $\omega_{ij}$ represents a two-dimensional element of a space vector matrix (*i, j*). When the index is greater than 0, it indicates that there is a positive spatial correlation between provinces. When the index is less than 0, it indicates that there is a negative spatial correlation between provinces. When the index is 0, there is no spatial correlation.

On this basis, the paper further constructs a spatial Durbin model to identify the spatial correlation between the *DE* and the *HDHI* formula (4).


(4) $$HDHI_{it}=\alpha_{0}+\rho W_{ij}DE_{it}+\overset{6}{\underset{k=2}{\sum }}\alpha_{1}X_{kit}+\overset{6}{\underset{k=2}{\sum }}\alpha_{2}W_{ij}X_{kit}+\varepsilon_{it}$$

$W_{ij}$ is the space vector weight matrix. ρ is the spatial autocorrelation coefficient of the explained variable. $\alpha_{2}=0$ , spatial Durbin model degenerates into spatial lag model (SAR). $\alpha_{2}+\rho \alpha_{1}=0$ , spatial Durbin model degenerates into spatial error model (SEM).

## 3 Data sources

### 3.1 Dependent variable: high-quality development of the healthcare industry

In the measurement of the HDHI, we have carried out measurement from four aspects, including medical service, drug service, technical service, and upgrade service. In this index system, a total of 12 indicators are selected for measurement, in order to reflect the HDHI in China. Since this paper chooses a multi-index measurement method, it is necessary to measure the comprehensive level. Therefore, this paper adopts entropy weight method to synthesize the indicators in the index system (Table 1).


(5) $$\mathrm{Positive}\;u_{ij}=\left(X_{ij}-\min X_{ij}\right)/\left(\max X_{ij}-\min X_{ij}\right).$$

(6) $$\mathrm{Negative}\;u_{ij}=\left(\max X_{ij}-X_{ij}\right)/\left(\max X_{ij}-\min X_{ij}\right)$$

(7) $$U_{j}=\overset{n}{\underset{i=1}{\sum }}w_{i}u_{ij}$$

The use of entropy weight method first needs to judge the attribute of the index, that is, whether the index is positive or negative. Secondly, standardize the index. Thirdly, the weight of the whole index system is obtained. Finally, the comprehensive score of the index is obtained by multiplying the standardized index value and the calculated index weight. For the specific calculation steps of entropy weight method, see formula (5)–(7).

**Table 1:** Evaluation index system of high-quality development of the healthcare industry.

First grade index	Second grade index	Third grade index	Attribute
High-quality development of the healthcare industry	Medical service	Number of tertiary level hospitals	+
		Number of health care institutions	+
		Number of beds in medical institutions	+
	Drug service	Production of chemical drugs	+
		Number of post offices	+
		Number of express	+
	Technical service	Number of patents granted by region	+
		R&D investment	+
		Number of health education training personnel of health education professional institutions	+
	Upgrade service	Number of travel agencies	+
		Number of beds for older people in nursing homes	+
		Number of tourism employees	+

Author collation.

### 3.2 Independent variable: digital economy

The independent variable is each province’s DE in China. The development of DE has always been centered on the internet, an essential carrier (28), and has a profound impact on economic growth in China. In the indicators of DE constructed in this paper, we focus on the two dimensions of internet development and digital transactions. According to existing studies (29), we select four indicators: the number of internet access users, the proportion of computer service and software employees in urban units, telecommunications traffic *per capita*, and the number of mobile phones among 100 people. Moreover, considering that digital transactions are booming in the era of DE, digital financial inclusion has become an important driving force for the development of DE (30). Therefore, the index of digital financial inclusion is added to the research. The above data are all from the statistical yearbook of China’s provinces. The digital financial inclusion comes from the Digital inclusive Finance Development index compiled by the Digital Finance Research Center of Peking University and Ant Financial Services Group^1^ (31). Through principal component analysis (PCA), the indexes were standardized and dimensionality reduced to obtain each province’s DE development level.

### 3.3 Control variables

Gross Domestic Product (GDP): GDP is one of the most representative indicators of economic growth. China’s huge economic scale is the basis for HDHI (32). Openness (OPEN): we use foreign direct investment to measure regional openness. Number of patents granted (PAT): technological innovation is an essential driving force to optimize industrial structure and promote economic development. HDHI cannot be separated from the technical support. Population size (POP): the number of permanent residents in each province; we also control the quadratic term of population size (POP^2^) to capture the nonlinear relationship. Urbanization (URB): the ratio of urban population to total population is measured in this paper. Table 2 shows descriptive statistics of variables.

**Table 2:** Descriptive statistics of variables.

*Variable*	*N*	*Mean*	*SD*	*Min*	*Max*
*HE*	300	0.651	0.539	0.165	0.856
*DE*	300	0.938	1.278	−1.023	6.958
*GDP*	300	2.484	2.054	0.137	11.115
*OPEN*	300	0.200	0.342	0.003	2.745
*PAT*	300	5.860	8.937	0.050	70.973
*POP*	300	45.998	28.378	5.680	126.240
*URB*	300	0.590	0.122	0.350	0.896

**Figure 1:**
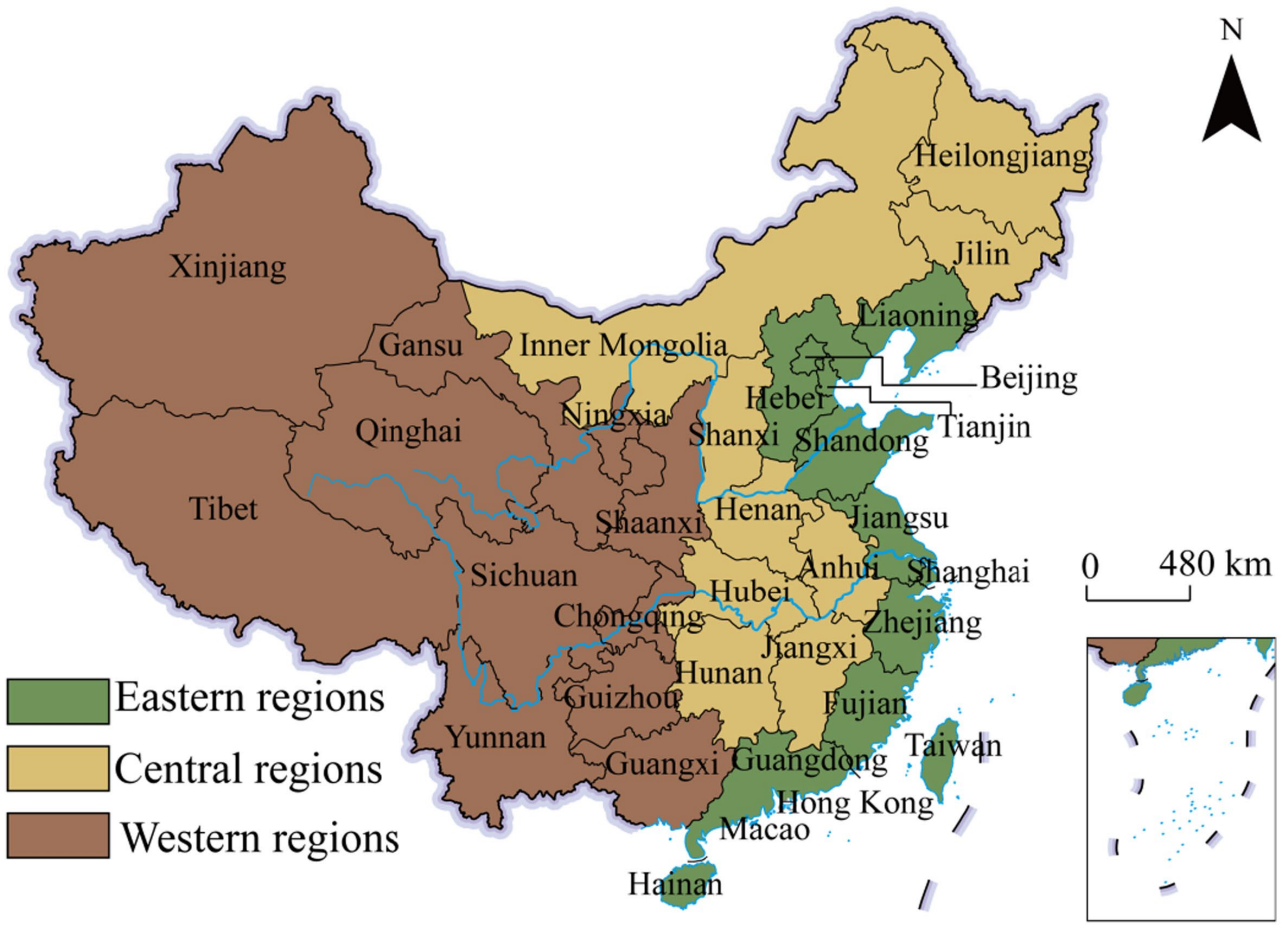
Study areas.

The research data in this paper come from China’s national statistical data, including China Statistical Yearbook, statistical yearbook, and statistical bulletin issued by Chinese provinces, China Health and Family Planning Statistical Yearbook, China Culture and Tourism Statistical Yearbook, etc. When there are missing values in the panel data we construct, we supplement the missing data with interpolation method to ensure that the panel data we construct is a balanced one. Due to the limitation of research data, the research period of this paper is selected from 2011 to 2020. The research region covers 30 provinces (autonomous regions and municipalities directly under the Central Government) in China, among which Tibet, Hong Kong Special Administrative Region, Macao Special Administrative Region, and Taiwan Province were not included in the study sample due to lack of data (Figure 1).

## 4 Empirical results

### 4.1 Baseline regression analysis

This part is based on the econometric model (1) set above (Section 2.1), and Stata 17.0 software is used to calculate and analyze the data. In the baseline regression of Table 3, column (1) is listed as the regression result only included two-way fixed effect (province fixed effect and year fixed effect). Based on the regression results, we find a significant positive relationship between DE and HDHI. We include all the control variables and no longer control the fixed effect in column (2). We find that the regression result still has a significant positive relationship. Based on column (2), we add the fixed effect of the year in column (3) and the fixed effect of the province in column (4), and the result remains robust. We include all control variables and fixed effects and adopt more rigorous robust standard error statistics in column (5). The results are still positive and significant, indicating that DE significantly positively affects HDHI.

**Table 3:** Baseline regression results.

	(1)	(2)	(3)	(4)	(5)
	*HC*	*HC*	*HC*	*HC*	*HC*
*DE*	0.632^***^ (0.116)	0.511^***^ (0.112)	0.566^***^ (0.166)	0.684^***^ (0.126)	0.671^***^ (0.140)

*GDP*		0.566^***^ (0.192)	0.725^***^ (0.204)	−0.114^*^ (0.064)	−0.109 0.083
	
*OPEN*		−0.401 (0.465)	−0.310 (0.469)	0.011 (0.116)	0.013 (0.126)
	
*PAT*		−0.081^***^ (0.021)	−0.079^***^ (0.016)	0.004 (0.010)	0.004 (0.011)
	
*POP*		0.029^*^ (0.015)	0.025 (0.015)	0.183^***^ (0.026)	0.175^***^ (0.031)
	
*POP^2^*		−0.000 (0.000)	0.000 (0.000)	−0.000^**^ (0.000)	−0.000^**^ (0.000)
	
*URB*		4.098^***^ (1.236)	4.671^***^ (1.410)	−1.798 (1.513)	−1.798 (1.568)
	
*Constant*	3.174^***^ (0.419)	−0.776 (0.793)	−1.619^*^ (0.945)	2.283 (1.440)	2.283 (1.675)

*Year F. E.*	Yes	No	Yes	Yes	Yes
*Province F. E.*	Yes	No	No	Yes	Yes
*Observations*	300	300	300	300	300
*R^2^*	0.914	0.469	0.563	0.917	0.925

(Robust) Standard errors are shown in parentheses, ^*^, ^**^, and ^***^ stand for statistical significance at the 0.1, 0.05, and 0.01 significance levels, respectively.

### 4.2 Robustness test

#### 4.2.1 Robustness test I: replace independent variables

The principal component analysis is used to construct the index system for independent variables in baseline regression. In order to avoid the interference of weight on the results, we weighted the indicators in the benchmark regression by a new weight calculation method to obtain the development level of DE calculated based on different methods in this section. We combine the analytic Hierarchy Process (AHP), entropy weight method (EVM), and least squares decision (LSD) model to control further the randomness and inaccuracy of index weight calculation (33). The weight calculation method can control the deviation of weight calculation in a minimum range (34). The specific calculation method is as follows formula (8).


(8) $$\min H\left(w\right)=\overset{m}{\underset{i=1}{\sum }}\overset{n}{\underset{j=1}{\sum }}\left\{{\left[\left(u_{j}-w_{j}\right)X_{ij}\right]}^{2}+{\left[\left(v_{j}-w_{j}\right)X_{ij}\right]}^{2}\right\}$$

where subjective weight vector *v* = (*v*_1_, *v*_2_,…, *v*_n_)T, objective weight vector *u* = (*u*_1_, *u*_2_,…, *u*_n_)^T^, integrated weight vector w = (*w*_1_, *w*_2_,…, *w*_n_)T, $\overset{n}{\underset{i=1}{\sum }}w_{i}=1$ , *w_i_* ≥ 0 (*i* = 1,2,…,*n*).

In Table 4, column (1) lists the development level of *DE* obtained by changing the weight calculation method. The relationship between DE and HDHI is still positively significant. This indicates that the weight calculation method does not affect the conclusion of baseline regression.

**Table 4:** Robustness test.

	(1)	(2)	(3)	(4)	(5)	(6)
	*CE*	*CE*	*CE*	*CE*		
*L.CE*			1.058^***^ (0.009)	0.934^***^ (0.037)		
				
*DE*	0.574^***^ (1.231)	0.654^***^ (0.126)	0.043^***^ (0.014)	0.029^***^ (0.002)	0.771^***^ (0.149)	0.369^***^ (0.093)

*Controls*	Yes	Yes	Yes	Yes	Yes	Yes
*Year F. E.*	Yes	Yes			Yes	Yes
*Province F. E.*	Yes	Yes			Yes	Yes
*AR*(1)			0.035	0.035		
*AR*(2)			0.233	0.244		
*Sargan test*			0.453	0.255		
*Observations*	300	300	270	240	260	260
*R^2^*	0.983	0.313			0.984	0.992

Standard errors are shown in small parentheses, ^*^, ^**^, and ^***^ stand for statistical significance at the 0.1, 0.05, and 0.01 significance levels, respectively.

#### 4.2.2 Robustness test II: change different estimation methods

In the previous article, ordinary least square (OLS) method tests the relationship between DE and HDHI. Although the OLS method has advantages (e.g., simple calculation principle, convenient calculation, and fast computing speed), it is also subject to the interference of many problems (e.g., endogeneity, heteroskedasticity). Therefore, we replace the estimation method used in the baseline regression analysis and use the First-order Differential Generalized Method of Moments (GMM) and System Differential GMM for the robustness test. We use the least square dummy variable method (LSDV) to control the fixed effects in baseline regression. We choose the intra-group transformation method to control the fixed effects of province and year. The estimated results are reported in column (2) of Table 4. Moreover, First-order Differential GMM and System Differential GMM are adopted for estimation (35). The regression results are reported, respectively, in columns (3) and (4) of Table 4, which show that the difference in estimation methods does not lead to the change in baseline results.

#### 4.2.3 Robustness test III: subsample regression test

In order to avoid the effects that special samples have on the regression results, we remove special provinces (municipalities directly administered by the Central government and ethnic minority regions) from the database. This is because municipalities directly administered by the central government of China have more policy support, while Chinese ethnic minorities inhabit autonomous regions. In order to support the development of ethnic minority regions, the central government of China adopts specific supportive policies. Although municipalities and autonomous regions are at the same administrative level as provinces, they differ regarding development mode and policy support. This could interfere with the research results of this paper. Therefore, we refer to existing studies (36), excluded the samples of Beijing, Shanghai, Tianjin, and Chongqing, and excluded the samples of Xinjiang, Ningxia, Inner Mongolia, and Guangxi autonomous regions, respectively. The regression results are reported in columns (5) and (6) of Table 4.

#### 4.2.4 Robustness test VI: instrumental variable estimation

In baseline regression, the results may be biased due to the omitted variable, which is challenging to observe or measure. Moreover, the regression model may have the problem of reverse causation. This is because the HDHI needs to rely on information, digitalization, and intelligence. Therefore, the HDHI will force the region to enhance the development of the DE. Therefore, we adopt instrumental variable to alleviate the endogenous problems.

There are two conditions (relevance and exclusion) for using instrumental variable. We choose the geographical distance from the capital city to Hangzhou as the instrumental variable. On the one hand, Hangzhou is the origin of China’s DE with a high level of development of DE (30), which gather many internet enterprises represented by Alibaba, NetEase, and Byte dance. The region near Hangzhou is more likely to be affected by the development of DE in Hangzhou, which satisfies the relevance of instrumental variable. On the other hand, geographical distance is an exogenous variable (37), which has no direct impact on HDHI. Geographical distance is the cross-section datasets, so fixed effects in the model absorb it. Therefore, we multiply it with the number of internet employees to get the interaction term as the instrumental variable. Moreover, we also report the estimated results of constructing interaction terms using spherical distance.

We find that the Anderson canon. Corr. LM statistics significantly reject the null hypothesis, showing that there is not insufficient identification, based on instrumental variable estimation findings shown in Table 5. An endogenous variable and an instrumental variable are correlated. The existence of weak instrumental variables is strongly rejected, and the Crag-Donald Wald F statistic is much greater than the Stock-Yogo weak ID test critical values. It is shown that the estimation contains no weak instrumental variables. The robustness of the baseline regression is demonstrated by the estimated results, which indicate a considerable beneficial influence of DE on HDHI.

**Table 5:** Estimation results of instrumental variables.

	(1)	(2)	(3)	(4)
	*HC*	*HC*	*HC*	*HC*
*DE*	0.318^*^ (0.154)	0.925^***^ (0.314)	0.327^*^ (0.177)	0.968^***^ (0.332)
*Controls*	Yes	Yes	Yes	Yes
*Year F. E.*	No	Yes	No	Yes
*Province F. E.*	No	Yes	No	Yes
*Observations*	300	300	300	300
*Anderson canon. corr. LM statistic*	118.459 [0.000]	72.435 [0.000]	118.355 [0.000]	72.345 [0.000]
*Cragg-Donald Wald F statistic*	189.32	79.325	192.39	78.326
*Stock-Yogo weak ID test critical values: 10% maximal IV size*	16.38	16.38	16.38	16.38
The first-stage regression results				
	(5)	(6)	(7)	(8)
	*DE*	*DE*	*DE*	*DE*
*IV*	2.756^***^ (0.295)	1.985^***^ (0.321)	2.925^***^ (0.205)	1.963^***^ (0.217)
*Controls*	Yes	Yes	Yes	Yes
*Year F. E.*	No	Yes	No	Yes
*Province F. E.*	No	Yes	No	Yes
*Observations*	300	300	300	300
*R^2^*	0.769	0.985	0.719	0.983

Standard errors are shown in small parentheses, *p* value are shown in middle parentheses ^*^, ^**^, and ^***^ stand for statistical significance at the 0.1, 0.05, and 0.01 significance levels, respectively.

### 4.3 Heterogeneity test

#### 4.3.1 Heterogeneity of the development level of the digital economy

Geographical location can vary the identification results of causal effects (38–40). This part focuses on the differences in HDHI brought by different degrees of development of DE. We explore this heterogeneity based on the quantile regression model. The panel quantiles divide the data into five quantiles (5, 25, 50, 70, and 95th) to investigate the relationship between DE and HDHI. Based on the development level of DE heterogeneity’s estimation results in Table 6, we find that the positive effect of DE on HDHI is significantly below the 50-quantile level. The relationship between DE and HDHI above the 50-quantile level is positive but insignificant. The HDHI of the DE presents an inverted U-shaped trend of first increasing or decreasing below the 50-quartile level. The results provide exciting and unique policy implications.

**Table 6:** Estimation results of heterogeneity of developmental level of digital economy.

	Q5	Q25	Q50	Q75	Q95
	*HC*	*HC*	*HC*	*HC*	*HC*
*DE*	0.211^***^ (0.047)	0.267^***^ (0.042)	0.233^**^ (0.100)	0.371 (0.233)	0.219 (0.424)
*Controls*	Yes	Yes	Yes	Yes	Yes
*Constant*	−1.962^***^ (0.349)	−1.053^***^ (0.297)	−0.765 (0.733)	−1.787 (1.499)	−3.867 (3.507)
*Observations*	300	300	300	300	300
*Pseudo R^2^*	0.463	0.365	0.333	0.325	0.310

Standard errors are shown in small parentheses, ^*^, ^**^, and ^***^ stand for statistical significance at the 0.1, 0.05, and 0.01 significance levels, respectively.

#### 4.3.2 Regional heterogeneity of provinces

China has a vast territory. Different regions have considerable differences in factor endowment, industrial institutions, and economic base (41–43). The impact of DE on HDHI may vary systematically depending on the development level and region of DE (44–46). First of all, we divide China’s geographical areas into two dimensions: east–west horizontal and north–south vertical. For relevant standards, see (29). Based on the estimation results of the regional heterogeneity in Table 7, we find that the DE significantly promote HDHI in the eastern and central-western regions. However, the effect of the DE on the HDHI will be stronger in the eastern regions than in the central-western regions. Compared with the northern region, the DE has a significant impact on HDHI in the southern region. However, the DE has not had a significant impact on the HDHI in the northern region.

**Table 7:** Estimation results of regional heterogeneity of provinces.

	Eastern region	Central-western regions	Northern region	Southern region
	*HC*	*HC*	*HC*	*HC*
*DE*	0.614^***^ (0.136)	0.587^**^ (0.211)	0.069 (0.235)	0.843^***^ (0.168)

*Constant*	3.657^*^ (1.884)	−3.596^**^ (1.616)	−5.449^***^ (1.787)	−0.339 (1.628)

*Controls*	Yes	Yes	Yes	Yes
*Year F. E.*	Yes	Yes	Yes	Yes
*Province F. E.*	Yes	Yes	Yes	Yes
*Observations*	110	190	150	150
*R^2^*	0.584	0.468	0.498	0.392

Standard errors are shown in small parentheses, ^*^, ^**^, and ^***^ stand for statistical significance at the 0.1, 0.05, and 0.01 significance levels, respectively.

### 4.4 Spatial econometric model analysis

#### 4.4.1 Spatial autocorrelation test

Ignoring the spatial effects of each variable in the research may cause bias in the estimation results, so it is necessary to analyze the spatial correlation of each variable before setting the model. The global Moran’s I for the high-quality development of the DE and the health industry is significantly positive at the level of 1%, rejecting the null hypothesis that there is no spatial autocorrelation. The relevant estimates are presented in Table 8.

**Table 8:** Global Moran’s I of digital economy and high-quality development of the healthcare industry.

Year	Moran’ s I	Variance	*Z*-score	*p* value
2011	0.325/0.456	0.008/0.012	4.125/4.698	0.000/0.000
2012	0.416/0.469	0.009/0.011	4.269/4.879	0.000/0.000
2013	0.459/0.519	0.007/0.019	4.375/5.102	0.000/0.002
2014	0.496/0.568	0.008/0.014	4.444/5.329	0.000/0.000
2015	0.516/0.614	0.008/0.018	4.958/5.598	0.000/0.001
2016	0.558/0.625	0.006/0.021	5.021/5.981	0.000/0.000
2017	0.579/0.646	0.008/0.017	5.264/6.108	0.000/0.000
2018	0.654/0.692	0.007/0.012	5.569/6.541	0.000/0.002
2019	0.713/0.749	0.009/0.014	5.987/6.894	0.000/0.000
2020	0.815/0.796	0.007/0.019	6.1977.102	0.000/0.004

#### 4.4.2 Spatial Durbin model

LM test was used to judge the model, and the LM statistic coefficients of SEM and Robust LM statistic coefficients were 6.215 and 11.065, respectively. The LM statistic coefficients of SAR and Robust LM statistic coefficients are 10.235 and 14.269, respectively, and the four variables are all significant at 1% confidence level. Therefore, the Spatial Durbin Model is required for spatial effect estimation.

The SAR, SEM, and SDM regression results are reported in Table 9. The regression results of SDM model show that the spatial spillover effect coefficient of DE on the HDHI is 0.612, which is statistically significant at 1% level. In the economic sense, it means that the healthcare industry has obvious agglomeration spillover effect in space, and the high-quality development of the local healthcare industry will drive the HDHI in the neighboring region. The estimated coefficient of W*DE is 0.597, which is statistically significant at 1% level. In an economic sense, for every 1% increase in the level of high-quality development of the local healthcare industry, the healthcare industry in the neighboring region will increase by 0.597%.

**Table 9:** Spatial spillover effect regression results.

	SAR	SEM	SDM
	*HC*	*HC*	*HC*
W*DE			0.597^***^ (0.211)
		
*DE*	0.526^***^ (0.005)	0.594^***^ (0.091)	0.614^***^ (0.165)

*rho*	0.564^***^ (0.015)		0.612^***^ (0.085)
	
*lambda*		0.621^***^ (0.169)	
		
*LR test*	13.564^***^	12.678^***^	
*Wald test*	14.635^***^	15.658^***^	
*R^2^*	0.841	0.859	0.965

Standard errors are shown in small parentheses, ^*^, ^**^, and ^***^ stand for statistical significance at the 0.1, 0.05, and 0.01 significance levels, respectively.

#### 4.4.3 Spatial spillover effect decomposition

Since the SDM model includes the spatial vector weight matrix, the feedback effect of the spatial lag term may be brought in the recognition, and the estimation results of the model cannot be accurately recognized. Therefore, in order to explain the estimation results of SDM model more scientifically and reasonably, partial differential method is used to calculate the total utility, direct effect, and indirect effect of each variable (47–49). According to the decomposition results, the estimated coefficients of total utility, direct effect, and indirect effect are 0927, 0.623, and 0.601, respectively, and are statistically significant at 1% level. This means that the development of the DE will significantly drive the HDHI in the overall region, the local region and the neighboring region.

## 5 Conclusion and policy implications

### 5.1 Conclusion

In the rapid development of DE, this paper focuses on the DE enabling HDHI. We empirically studied the relationship between DE and HDHI based on panel data from 30 Chinese provinces from 2011 to 2020. The main conclusions are as follows:

We find that DE significantly positively affects HDHI. This result is robust after a series of robustness tests (e.g., replace independent variables, change different estimation methods, and sub-sample regression test). The results also remain robust to overcome the endogenous problem.
Heterogeneity analysis shows that the impact of DE on the HDHI is only significant below the median. Regional heterogeneity analysis shows that the impact of DE on the HDHI is more significant in the eastern and southern regions.
The results of spatial econometric analysis show that the development of DE has a significant spatial spillover effect on the HDHI. The development of DE in this region will significantly promote the high-quality development level of healthcare industry in neighboring regions.

### 5.2 Policy implications

China should prioritize the advancement of coordinated and balanced development of DE across various regions, while also striving to reduce the digital divide that exists across these regions. China should strategically align the growth of DE between the eastern and central-western regions, as well as between the southern and northern regions. The Chinese government ought to actively encourage the development of digital infrastructure in underdeveloped regions and facilitate the widespread adoption of digitalization efforts across all regions.

China should prioritize the implementation of well-designed high-quality development plans and guidelines for the healthcare business, as well as for the growth of DE, in order to steer development in a scientifically planned manner. The implementation of classified policies, addressing the health care gap resulting from the development of the digital economy, providing targeted attention to various industrial actors, and establishing the operational mechanism and development model of the health care industry ecosystem in the context of digitalization are imperative.

### 5.3 Future research

This study uses the data at the provincial level of China, but we do not study at the global, national, prefecture, county, and household scales. We believe that we can find some interesting conclusions.

In this paper, reduced form estimation method is adopted. In the future, structural form estimation method can be considered to estimate the relationship between DE and HDHI, in order to get more scientific and reasonable conclusions.

This paper uses the index system method to measure DE. Some exogenous impacts can be considered. For example, China’s digital city construction and big data pilot zones will provide us with quasi-natural experiments.

## Data availability statement

The datasets presented in this study can be found in online repositories. The names of the repository/repositories and accession number(s) can be found below: https://idf.pku.edu.cn/yjcg/zsbg/513800.htm.

## Author contributions

ZD: Writing – original draft. XQ: Writing – review & editing. CL: Writing – review & editing.

## Footnotes

^1^See https://idf.pku.edu.cn/yjcg/zsbg/513800.htm
